# The complete chloroplast genome of *Scutellaria meehanioides* (Lamiaceae) from Shaanxi Province, China

**DOI:** 10.1080/23802359.2021.1927877

**Published:** 2021-05-19

**Authors:** Caijuan Zhang, Pengguo Xia, Rui Wu, Dennis Mans

**Affiliations:** a Key Laboratory of Plant Secondary Metabolism and Regulation of Zhejiang Province, College of Life Sciences and Medicine, Zhejiang Sci-Tech University, Hangzhou, China; b Zhejiang Provincial Key Laboratory of Resources Protection and Innovation of Traditional Chinese Medicine, Zhejiang A&F University, Hangzhou, China; c Faculty of Medical Sciences, Anton de Kom University of Suriname, Paramaribo, Suriname

**Keywords:** Chloroplast genome, Lamiaceae, phylogenetic analysis, *Scutellaria meehanioides*

## Abstract

*Scutellaria meehanioides* C.Y.Wu is a medicinal perennial herb native to China. The complete chloroplast genome sequence of the *S. meehanioides* was determined and assembled using next generation sequencing methodologies. The complete genome is 152,484 base pairs (bp) in length and has an overall GC content of 38.4%. The chloroplast genome contains, a large single-copy region (LSC) of 83,859 bp, small single-copy region (SSC) of 17,467 bp and a pair of inverted repeats (IRs) of 24,029 bp. The genome of *S. meehanioides* contains 133 genes, including 87 protein-coding, 38 tRNA,and 8 rRNA genes. Phylogenetic analysis fully resolved *S. meehanioides* in a clade with *S. orthocalyx.* This study provides useful information for future genetic study of *S. meehanioides*.

*Scutellaria* L. is species-rich, with more than 300 species currently accepted and widely distributed in Europe, the United States and East Asia (Zhang et al. 1998). Some species in *Scutellaria* have been used as local medicine for thousands years (Shang et al. 2010). *Scutellaria meehanioides* was one of them which is a perennial herb classified in the Lamiaceae and mainly distributed in Shaanxi Province, China . The root of *S. meehanioides* was used as a crude medicine for antitumor, anti-oxidant and hepato-protective (Yang et al. 2020). In this study, we report the complete chloroplast genome of *S. meehanioides*, which revealed the phylogenetic relationship with other species in *Scutellaria* and the Lamiaceae.

The samples of *S. meehanioides* were collected from Mt. Wutai, Xi'an City, Shaanxi Province (33°59′18.45″N, 108°58′23.44″E). The voucher specimen was deposited in XBGH (The Herbarium of Xi’an Botanical Garden, http://www.xazwy.com） (Voucher number: *Xun Lulu* et al.*00918,* Lulu Xun, xunlulu20032006@126.com). The total genomic DNA was extracted from fresh leaves using the CTAB extraction method (Doyle and Doyle 1987). The genomic library was sequenced using the Illumina NovaSeq platform. Raw PE reads of about 2.4 Gb were trimmed and filtered, using fastp application (Chen et al. 2018), resulting in about 2.38 Gb clean PE reads. NOVOplasty v2.7.2 (Dierckxsens et al. 2016) was used for the de novo assembly of chloroplast genome using *S. baicalensis* (GenBank accession no. MF521663) as the seed reference and the parameters ‘Type = chloro K-mer = 39 Genome range = 13,000–17,000’. The sequence was annotated using GeSeq (https://chlorobox.mpimp-golm.mpg.de/geseq-app.html) and visually checked in Geneious v11.0.3 (Kearse et al. 2012) using the chloroplast genome of *S. tsinyunensis* (GenBank accession: NC 050761) as a reference. The complete chloroplast genome of *S. meehanioides* is publicly available under GenBank accession number MW381011.

The complete chloroplast genome of *S. meehanioides* is 152,484 bp in length and has an overall GC content of 38.4%, consisting of a small single-copy region (SSC) of 17,467 bp, a pair of inverted repeat regions (IRa and IRb) with the same length of 24,029 bp, and a large single-copy region (LSC) of 83,859 bp. One hundred thirty-three genes were predicted, including 87 protein-coding, 38 tRNA, and 4 rRNA genes.

To confirm the phylogenetic position of *S. meehanioides*, the maximum likelihood (ML) phylogenetic analyses were conducted by IQTREE v1.6.7 (Nguyen et al. 2015) based on 20 complete chloroplast genome sequences of Lamiaceae, under TVM + F+R3 model with 5000 bootstrap replicates. The phylogenetic tree revealed that *S. meehanioides* was closely related to *S. orthocalyx* (Figure 1). Some plastid genes, such as matK-trnK, atpB-atpE, psbC-psbD, and rps3-rpl22, overlap each other in the Lamiaceae chloroplast genomes (Liang et al. 2019). The newly characterized *S. meehanioides* complete chloroplast genome provides a reference for the phylogenetic relationships and assessment of the genetic structure of *Scutellaria* and the Lamiaceae.

**Figure 1.**
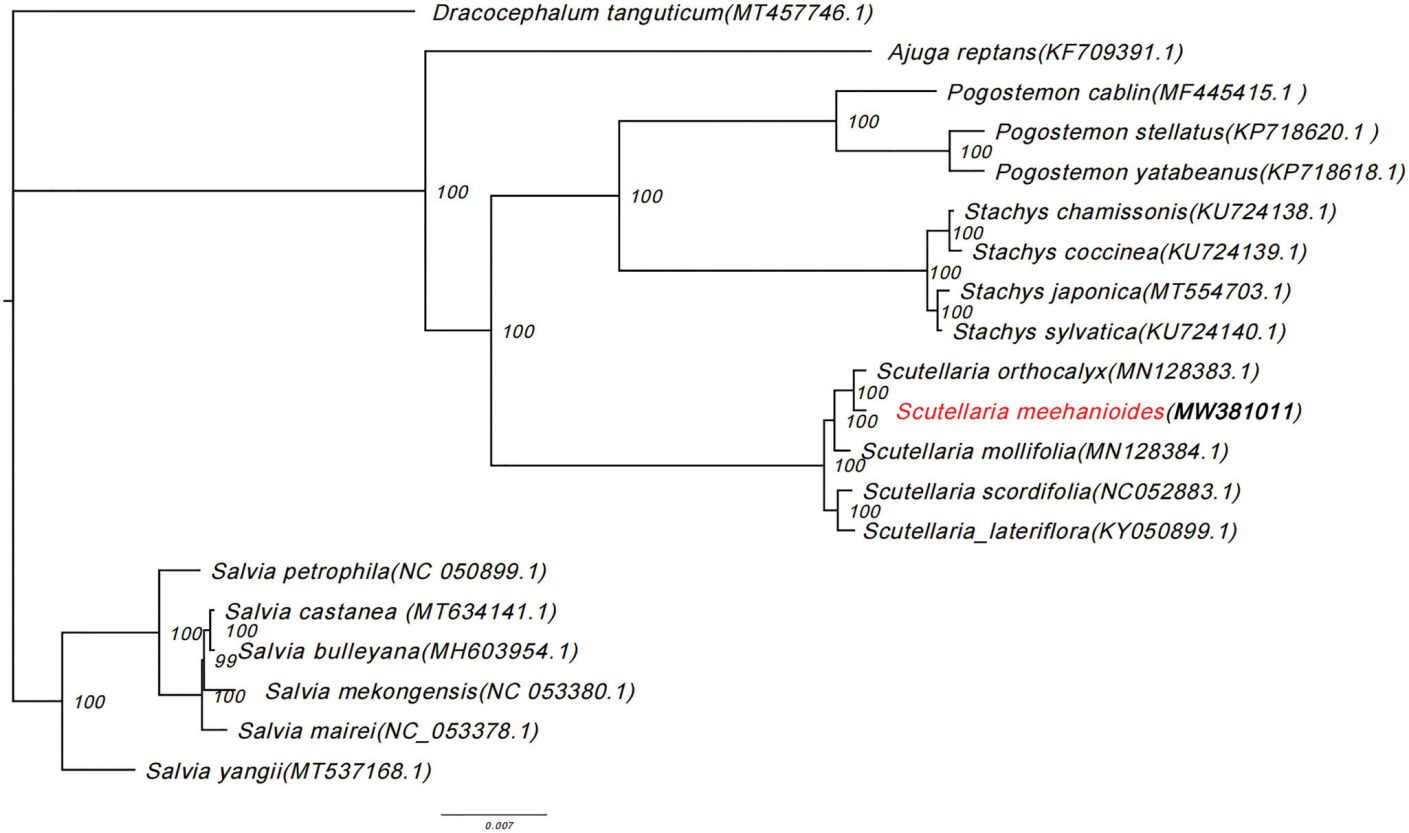
Phylogenetic tree showing the relationship between *Scutellaria meehanioides* and 19 Lamiaceae species. Phylogenetic tree was constructed based on the complete chloroplast genomes using maximum likelihood (ML) with 5000 bootstrap replicates. Numbers in each node indicate the bootstrap support values.

## Data Availability

The genome sequence data that support the findings of this study are openly available in GenBank of NCBI (https://www.ncbi.nlm.nih.gov) under the Accession no. MW381011. The associated BioProject, SRA, and Bio-Sample numbers are PRJNA715065, SRR13987450, and SAMN18325271, respectively.
